# The Plastid Casein Kinase 2 Phosphorylates Rubisco Activase at the Thr-78 Site but Is Not Essential for Regulation of Rubisco Activation State

**DOI:** 10.3389/fpls.2016.00404

**Published:** 2016-03-31

**Authors:** Sang Y. Kim, Kyle W. Bender, Berkley J. Walker, Raymond E. Zielinski, Martin H. Spalding, Donald R. Ort, Steven C. Huber

**Affiliations:** ^1^Global Change and Photosynthesis Research Unit, United States Department of Agriculture – Agricultural Research Service, UrbanaIL, USA; ^2^Plant Biology, University of Illinois at Champaign–Urbana, UrbanaIL, USA; ^3^Carl R. Woese Institute for Genomic Biology, UrbanaIL, USA; ^4^Genetics, Development and Cell Biology, Iowa State University, AmesIA, USA

**Keywords:** Rubisco, Rubisco activase, redox regulation, protein phosphorylation, modification-specific antibodies

## Abstract

Rubisco activase (RCA) is essential for the activation of Rubisco, the carboxylating enzyme of photosynthesis. In *Arabidopsis*, RCA is composed of a large RCAα and small RCAβ isoform that are formed by alternative splicing of a single gene (*At2g39730*). The activity of Rubisco is controlled in response to changes in irradiance by regulation of RCA activity, which is known to involve a redox-sensitive disulfide bond located in the carboxy-terminal extension of the RCAα subunit. Additionally, phosphorylation of RCA threonine-78 (Thr-78) has been reported to occur in the dark suggesting that phosphorylation may also be associated with dark-inactivation of RCA and deactivation of Rubisco. In the present study, we developed site-specific antibodies to monitor phosphorylation of RCA at the Thr-78 site and used non-reducing SDS-PAGE to monitor the redox status of the RCAα subunit. By immunoblotting, phosphorylation of both RCA isoforms occurred at low light and in the dark and feeding peroxide or DTT to leaf segments indicated that redox status of the chloroplast stroma was a critical factor controlling RCA phosphorylation. Use of a knockout mutant identified the plastid-targeted casein kinase 2 (*cpCK2α*) as the major protein kinase involved in RCA phosphorylation. Studies with recombinant cpCK2α and synthetic peptide substrates identified acidic residues at the –1, +2, and +3 positions surrounding Thr-78 as strong positive recognition elements. The *cpck2* knockout mutant had strongly reduced phosphorylation at the Thr-78 site but was similar to wild type plants in terms of induction kinetics of photosynthesis following transfer from darkness or low light to high light, suggesting that if phosphorylation of RCA Thr-78 plays a direct role it would be redundant to redox regulation for control of Rubisco activation state under normal conditions.

## Introduction

Rubisco activase (RCA) is a member of the large AAA^+^ super family of proteins (Hanson and Whiteheart, 2005) that uses the energy of ATP hydrolysis to function as a molecular chiropractic protein to allow the removal of inhibitory phosphate-esters from the Rubisco active site (Parry et al., 2008). Early *in vitro* studies demonstrated that RCA catalyzed the ATP-dependent activation of Rubisco, and also recognized that spontaneous Rubisco deactivation occurred concomitantly with RCA-mediated activation (Robinson et al., 1988). Thus, Rubisco activation state represents a dynamic equilibrium between activation and deactivation, and therefore RCA action is required to not only initiate but also maintain Rubisco activity. *In vivo*, the activity of Rubisco is controlled by RCA to coordinate the rate of electron transport and thereby maintain balance between RuBP synthesis and the rate of carboxylation (Salvucci et al., 1985).

The coordination between Rubisco activity and light level involves post-translational modifications that control RCA activity as a result of ADP inhibition in relation to the redox status of the chloroplast stroma (Zhang and Portis, 1999). In *Arabidopsis*, RCA is encoded by one gene (*At2g39730*) that is alternatively spliced to form a large ∼46-kDa RCAα and small ∼43-kDa RCAβ isoform. The α- and β-isoforms are nearly identical in primary structure except that the α-isoform has a 28 amino-acid C-terminal extension that contains two cysteine residues (Cys-451 and Cys-470). These two Cys residues can be reversibly oxidized to form a disulfide that increases inhibition by ADP, such that at physiological ATP/ADP ratios, reduced RCAα is active while the oxidized form is inactive (Zhang and Portis, 1999). Using transgenic *Arabidopsis* expressing cDNAs encoding either RCAα (designated rwt46) or RCAβ (designated rwt43) in the *rca^-^* mutant background (Somerville et al., 1982) it was demonstrated *in vivo* that down regulation of Rubisco at low light occurs as a result of control of RCAα by redox changes in the chloroplast stroma (Zhang et al., 2002). The β-isoform is not affected by oxidation and expression of RCAβ alone in the *rca^-^* mutant background dramatically reduces the extent of Rubisco deactivation upon transfer of plants from high to low light (Zhang et al., 2002). In *Arabidopsis*, there are roughly equal amounts of the α- and β-isoforms and it is known that heterooligomers form resulting in the native RCA protein being fully redox regulated.

In addition to redox regulation, there is also evidence for phosphorylation of RCA. In the original report (Reiland et al., 2009), Thr-78 of RCA was reported to be phosphorylated in a phosphoproteomic screen of leaf proteins, and this phosphosite was detected in more of the biological replicates harvested in the dark compared to the light suggesting that phosphorylation may be light/dark regulated. Subsequently, the Thr-78 phosphosite was identified in several other phosphoproteomic screens (Reiland et al., 2011; Aryal et al., 2012; Wang et al., 2013) of *Arabidopsis* leaves and developing seeds (Meyer et al., 2012). In targeted studies, RCA phosphorylation was examined in *Arabidopsis* rosettes subjected to various treatments that would impact photosynthesis, including light versus dark and different concentrations of CO_2_ in the light. In their targeted study (Boex-Fontvieille et al., 2013), phosphorylation of Thr-78 and Ser-172 was detected but phosphorylation at the Thr-78 site was uniquely increased in the dark, suggesting that phosphorylation might contribute to the dark inactivation of RCA, and as a result, Rubisco deactivation.

While it is clear that phosphorylation of RCA at the Thr-78 site occurs *in vivo* and appears to be light/dark regulated, many aspects remain unclear. For example, the factor(s) that trigger the dark-induced phosphorylation are not clear, with redox of the chloroplast stroma being one possible factor. It is also not known whether both of the RCA isoforms are phosphorylated because the sequences surrounding the phosphosite are identical in the α- and β-isoforms. Further, the identity of the protein kinase that phosphorylates Thr-78 *in vivo* has not been established, and finally the functional impact (if any) of RCA phosphorylation remains to be determined. The present study was conducted to begin to address these questions. To facilitate our studies, we developed a modification-specific polyclonal antibody (anti-pT78 antibodies) to monitor RCA phosphorylated at the Thr-78 site by immunoblotting, and RCA migration on non-reducing SDS-PAGE to monitor intra-subunit disulfide bond formation in the α-isoform. Transgenic *Arabidopsis* expressing either the α- or β-isoform in the *rca^-^* mutant (Somerville et al., 1982) background were used to monitor post-translational modifications of each isoform in the absence of the other. These transgenic plants, designated rwt46 and rwt43 for plants expressing the large ∼46 kDa α-isoform, or small ∼43 kDa β-isoform, have been extensively used to study the *in vivo* function of the two subunits of RCA (Kim and Portis, 2005; Barta et al., 2010; Carmo-Silva and Salvucci, 2013). Collectively, our results establish a robust phosphorylation of both isoforms in the dark, but at least in wild type *Arabidopsis* plants, redox regulation is sufficient to down regulate Rubisco activity upon transfer of plants from high light to low light.

## Materials and Methods

### Plant Growth

Transgenic *Arabidopsis* rwt43 plants, expressing only RCAβ, and rwt46 plants, expressing only RCAα, were produced and characterized previously (Zhang et al., 2002; Carmo-Silva and Salvucci, 2013). The cDNA constructs containing the α- or β-isoform of RCA were transformed into the *rca* mutant that has a single base change in the 5′-splice junction of intron 3, resulting in unspliced transcripts (Orozco et al., 1993) and no RCA protein (Zhang et al., 2002). Wild type *Arabidopsis thaliana*, ecotype Columbia-0, and transgenic (rwt43 and rwt46) seeds were sterilized with 10% (v/v) bleach at room temperature for 10 min. After sterilization, seeds were washed twice with 70% ethanol followed by distilled water. The seeds in distilled water were kept at 4°C for 3 days and then directly sown on soil. Long day and short day growth conditions were 16 h photoperiod and 8 h photoperiod, respectively, in growth chambers at 23°C with ambient CO_2_ and 150 μmol photons m^-2^s^-1^ photosynthetically active radiation (PAR).

The *Arabidopsis thaliana* accession Columbia T-DNA insertion line, GABI400A04, was obtained from the *Arabidopsis* Biological Research Center. In this line, the T-DNA insertion is in exon 5 of the gene. Homozygous plants were obtained by self-fertilization and selection with sulfadiazine for two generations and were confirmed by RT-PCR analysis as described below. Plants were grown as described above.

Relative growth rates were determined on plants grown with a short-day photoperiod following emergence by determining leaf area from chlorophyll fluorescence imaging (CF imager, Technologica Ltd, Colchester, UK). Plants were imaged three times a week until leaves began to overlap following 3 weeks of growth. Relative growth rate was determined from exponential fits of leaf area versus time and reported relative to total biomass (growth cm^2^ total cm^-2^ day^-1^) in order to minimize bias between individuals (Hoffmann and Poorter, 2002).

### Gene Expression Analysis

For RT-PCR analysis, RNA was extracted from 10 day-old-seedlings grown on MS plates using the RNeasy mini kit (Qiagen Inc., Valencia, CA, USA). Reverse transcription was performed with 1 μg of RNA using Superscript^®^ III Platinum^®^ One-Step qRT-PCR Kit (Life Technologies Corp, Grand Island, NY, USA). Primers used for amplification of ubiquitin, which was used as a positive control, were UBQ forward, 5′-GATCTTTGCCGGAAAACAATTGGAGGATGGT, and reverse, 5′-CGACTTGTCATTAGAAAGAAAGAGATAACAGG. For amplification of cpCK2, the primers were forward, 5′-GGCAGAATTCTATCATCCTGGG, and reverse, 5′-ATAAAAGAACGGCTCCTTGCGG.

### Transmission Electron Microscopy

Plants were sampled for transmission electron microscopy at the end of the 8-h photoperiod using a leaf punch and infiltrated with 2% glutaraldehyde buffered with 0.1 M sodium phosphate buffer (pH 6.8). Samples were serial dehydrated, critical point dried and embedded in LR white resin before sectioning using a diamond-bladed microtome. Samples were coated with gold–palladium before imaging using a JEOL transmission electron microscope (JEOL 2010 LaB6, Jeol USA) and analysis was performed in the Frederick Seitz Materials Research Laboratory at the University of Illinois.

### Recombinant Protein Production

The pET23 vector containing *Arabidopsis* RCAβcDNA lacking the transit peptide (residues 1 through 58) was provided by Dr. Rebekka Wachter and was transformed into BL21 (DE3) cells (Novagen, Gibbstown, NJ, USA) by heat shock. For protein expression, LB cultures containing the appropriate anti-biotic were inoculated with a 1:100 volume of saturated overnight culture and were grown at 37°C to an OD_600_ of 0.6 at which point cultures were induced with 0.3 mM IPTG at room temperature for 16 h. The recombinant protein was purified using Ni-NTA agarose (nitrilotriacetic acid; Qiagen) and the resin-bound protein was eluted in buffer containing 0.5 M imidazole. The eluted protein was dialyzed against a 1,000x volume of buffer (20 mM MOPS-NaOH, pH7.5, and 1 mM DTT) overnight in a cold room. The purified protein concentrations were measured by the Bradford assay using BSA as standard.

The full length *cpCK2* (At2g23070) cDNA clone was obtained from the *Arabidopsis* Biological Resource Center and a fragment lacking the transit peptide (residues 1–54) was subcloned into pET28a (Novagen) for recombinant protein production. The pET28a expression clone produced a protein consisting of residues 55 to 432 and a predicted molecular weight of 45.3 kDa (including the C-terminal 6xHis tag). Prior to PCR-based cloning, the *cpCK2* cDNA was subjected to PCR mutagenesis using the cpCK2ΔNcoI and cpCK2ΔNcoIrc primers to remove the internal NcoI restriction site. Mutagenized plasmid was recovered and then PCR amplified using the cpCK2NcoIF and cpCK2XhoIRprimers. Primers used for directed mutagenesis and cloning are indicated in Supplementary Table S1. PCR product and empty pET28a were restriction digested with NcoI and XhoI and digested fragments were gel purified. Purified digest products were ligated using Quick ligase (NEB) and 2 μl of ligation reaction was used to transform NEB5a (New England Biolabs, Ipswich, MA, USA) chemical competent cells. Transformation reactions were plated onto LB-agar containing 50 μg ml^-1^ kanamycin. Plasmids were isolated from kanamycin-resistant colonies and sequences of the inserts were confirmed by DNA sequencing. In-frame fusion with the C-terminal 6xHis tag was confirmed by *in silico* translation. Confirmed plasmids were transformed into the BL21(DE3) (New England Biolabs) expression host for recombinant protein production.

The cpCK2-His_6_ protein was expressed as described above for RCAβ and was purified by gravity-flow procedures for Ni-NTA agarose as previously detailed (Bender et al., 2015). Briefly, lysates containing cpCK2-His_6_ were adjusted to 300 mM NaCl and 50 mM imidazole and were bound to 500 μl of Ni-NTA agarose by gravity flow. Crude lysate was passed over the column five times for binding. The column was washed with 40 resin volumes of ice-cold binding buffer (50 mM Tris-HCl, pH 7.5, 300 mM NaCl, 60 mM imidazole), followed by 40 resin-volumes of binding buffer containing 0.25% (v/v) Triton X-100, and a further 40 resin-volumes of binding buffer. Bound protein was eluted in four 500 μl fractions of binding buffer supplemented with 500 mM imidazole. A sample of the unbound lysate and the eluted protein were assessed by SDS-PAGE to estimate the purity of cpCK2-His_6_ preparations, which was judged to be greater than 90% (data not shown). Purified protein was dialyzed against three 1000-fold volumes of 25 mM Tris-HCl pH 7.5, 100 mM NaCl prior to protein concentration determination and peptide kinase assays.

### Protein and Peptide Kinase Assays

Recombinant cpCK2 (kinase) and RCA (substrate) proteins were purified from *E. coli* and used for the substrate phosphorylation assays. Reaction mixtures contained 250 μM ATP, 10 mM MgCl_2_, 50 mM KCl, 20 mM Tris-HCl buffer (pH7.5), cpCK2 protein (0.1 μg), RCA protein (2 μg) in a total volume of 10 μL. Reactions were initiated by addition of RCA and allowed to proceed 10 min at room temperature unless specified otherwise. The kinase reactions were stopped by addition of one-third volume of 3X SDS loading buffer and the proteins were resolved in 10% SDS-PAGE gels, followed by immunoblotting with anti-pT78 antibodies to monitor phosphorylation of RCA at the Thr-78 site.

Kinetic analysis of cpCK2-His_6_ activity was carried out with synthetic peptide substrates using the P81 cellulose-binding method to monitor incorporation of ^32^P. Peptides based on the sequence surrounding RCA Thr-78 as well as peptide variants (Supplementary Table S2) were synthesized (GenScript, Piscataway, NJ, USA) based on residues 72 to 84 with an additional Arg residue added to the N-terminus to insure binding to the P81 paper.

Lyophilized peptides were dissolved in sterile water to a concentration of 10 mM and concentrations were confirmed by absorbance at 276 nm in 6 M guanidine HCl using an extinction coefficient of 1450 M^-1^cm^-1^ for a single Tyr residue. Kinase assay mixtures contained 40 mM Tris-HCl pH 7.5, 1 mM DTT, 10 mM MgCl_2_, 100 μM ATP, 3.7 × 10^-3^ MBq μl^-1^ [γ^32^P]ATP (2.5 Bq pmol^-1^; Perkin Elmer, Naperville, IL, USA), 0.5 μg of recombinant kinase and variable amounts of peptide substrate as indicated in figure captions. Final reaction volumes were 40 μl. Assays were carried out at room temperature for 10 min, after which 35 μl of each reaction was spotted on P81 cellulose paper. P81 papers were washed three times for 5 min each in 0.45% (v/v) ortho-phosphoric acid and ^32^P incorporation was assessed by liquid scintillation counting. Graphical analysis of the data and curve fitting for determination of *K*_m_ and V _max_ were carried out in Origin 2015 (OriginLab Corporation, Northampton, MA, USA). Experiments were repeated three times and values shown in **Figure 6** represent mean values with 95% CI of six technical replicates for each concentration of substrate peptide.

### DTT and H_2_O_2_ Feeding Assay

Leaves of 2-week-old *Arabidopsis* plants grown in a long day photoperiod were detached and submerged in 1.5 ml microfuge tubes containing water (control) or the indicated concentration of DTT or H_2_O_2_. The solutions were vacuum infiltrated into the tissue for 5 min using a water aspirator. The tubes were then transferred to light (100 μmol PAR m^-2^ s^-1^) or dark conditions for 1 h. The total proteins were extracted using 3X SDS sample buffer with or without 2-mercaptoethanol for the reducing and non-reducing SDS-PAGE condition, respectively.

### SDS-PAGE and Immunoblotting

Total protein was extracted from frozen leaf tissue by grinding in SDS-sample buffer containing 62.5 mM Tris-HCl, pH 8.0, 2% SDS, 1 M urea, 10% glycerol, and 0.005% bromphenol blue. For reducing conditions, an aliquot of the sample was supplemented with 2.1 M 2-mercaptoethanol, and for non-reducing conditions, an equivalent volume of water was added to another aliquot. Proteins were then resolved on 10% SDS-PAGE gels. Gel-resolved proteins were electrophoretically transferred to low fluorescence PVDF membrane (Immobilon-FL; from EMD Millipore, Kankakee, IL, USA) and treated with blocking solution (5% gelatin, Sigma) for 1 h at room temperature. Primary antibodies were diluted into PBST from a 1 mg ml^-1^ stock solution and used as follows: anti-RCA, 1:4000 dilution for 4 h; anti-pT78, 1:4000 dilution overnight; anti-His (Sigma), 1:3000 dilution overnight. The anti-RCA antibodies were produced against the peptide antigen: CELESGNAGEPAKLIR. Modification specific antibodies were generated against the phosphopeptide antigen: RGLAYDpT^78^DDQQDC (*Arabidopsis* anti-pT78). For each peptide antigen, the Cys residue at the N- or C-terminus was added for coupling to KLH. All custom antibodies were produced by GenScript (Piscataway, NJ, USA); for phosphopeptide antigens, the antibodies produced were sequentially affinity purified using the non-phosphorylated an then the phospho-containing antigen peptide. All primary antibody reactions were performed at RT. Immunoblots involving fluorescent secondary goat anti-rabbit antibodies (IRDye 800CW; LI-COR Biosciences, Lincoln, NE, USA) were diluted 1:10,000 in PBST and incubated for 1 h and were then scanned using a LI-COR Odyssey Infrared Imaging System for visualization. All immunoblotting experiments were performed at least twice and representative results are presented.

### Measurements of Photosynthetic Induction

Measurements of the photosynthetic induction were made on the youngest fully expanded leaves of 30–40 day-old-plants using a LI-COR 6400 XT fitted with a 2 cm^2^ chamber (LI-COR Biosciences, Lincoln, NE, USA, LI-COR Biosciences, 2010). Following a 20 min acclimation period (1000 μmol m^-2^ s^-1^ PAR), leaves were exposed to either darkness or low (30 μmol m^-2^ s^-1^ PAR) light for 30 min to de-activate Rubisco (Carmo-Silva and Salvucci, 2013). The photosynthetic induction was then measured following exposure to high light (1000 μmol m^-2^ s^-1^ PAR) for 30–60 min. All measurements were normalized against the photosynthetic rate following initial light acclimation immediately before the first dark or low light interval to correct for leaf-to-leaf variation in photosynthetic rate. Similar to past work, we did not see a shift in photosynthetic rates of *Arabidopsis* when values where corrected for the impact of stomatal conductance on CO_2_ availability and the uncorrected values are presented (Woodrow and Mott, 1989; Carmo-Silva and Salvucci, 2013). Values shown are mean values ± standard error of the mean from three experiments.

### Rubisco Activation State Assays

Activation state was measured using an activity assay of Rubisco before and after full chemical activation. To enable rapid sampling, leaf disks (0.6 cm^2^) were floated on a solution of 25 mM MES-NaOH, pH 5.5, flushed with ambient oxygen and CO_2_ (390 μmol mol^-1^). Leaf disks were illuminated with either 1000 or 30 μmol m^-2^ s^-1^ PAR using an LED lighting source. Following a 2-h acclimation period, disks were removed and flash frozen in liquid nitrogen. Initial Rubisco activity was assayed following rapid extraction at 4°C (50 mM HEPES-NaOH, pH 7.8, 1% polyvinylpolypyrrolidone, 1 mM EDTA, 10 mM DTT, 5 mM MgCl_2_, 0.1% Triton, and 1X Sigma–Aldrich plant protease inhibitor cocktail) using a glass homogenizer. Following homogenization, the extract was centrifuged at ∼15,000 × *g* for 20 s at 4°C and the supernatant assayed for initial activity spectrophotometrically from the enzymatically coupled conversion of NADH to NAD+ in reaction mixtures containing 100 mM EPPS-NaOH, pH 8.0, 10 mM MgCl_2_, 1 mM EDTA, 1mM ATP, 5 mM phosphocreatine, 20 mM NaHCO_3_, 0.2 mM NADH, and 0.5 mM RuBP with coupling enzymes: 25 U ml^-1^ creatine phosphokinase, 250 U ml^-1^ carbonic anhydrase, 25 U ml^-1^ 3-phosophoglycerate kinase, 20 U ml^-1^ glyceraldehyde-3-phosphate dehydrogenase, 20 U ml^-1^ glycerol-3-phosphate dehydrogenase, and at least 55 U ml^-1^ triosephosphate isomerase (Ruuska et al., 2000; Yamori and von Caemmerer, 2009; Carmo-Silva and Salvucci, 2013). The assay was optimized so that initial activity was measured ∼2 min after leaf disks were homogenized to minimize changes in the activation state. Final activity was determined following a 10-min activation period in the assay buffer and activation state determined as the ratio of initial to final activity. All assays and incubations were performed in the temperature-controlled multi-cuvette holder of the spectrophotometer maintained at 25°C. In initial optimizations, maximum Rubisco activity of extracts was found following 8 to13 min of activation.

## Results

### RCA Phosphorylation at the Thr-78 Site Is Light/Dark Regulated

**FIGURE 1:**
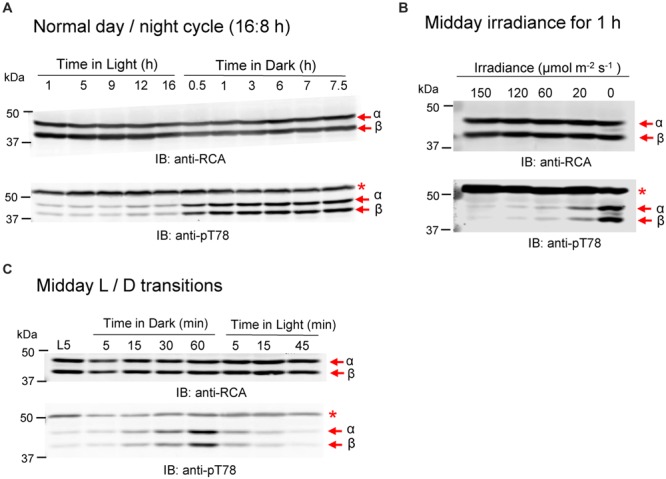
**Phosphorylation of RCA at the Thr78 site is light/dark regulated *in vivo*. (A)** Plants sampled at different times of the normal diurnal light/dark cycle. **(B)** Transfer of plants at midday to low light and darkness for 1 h. **(C)** Time course of a light-dark-light transfer of plants at midday. The experiment started 5 h after the beginning of the photoperiod (L5 sample). In each blot, the bands corresponding to the α- and β-isoforms are indicated. The red asterisk in the anti-pT78 blots indicates an off-target signal that serves as a loading control.

*Arabidopsis* leaves contain roughly equal amounts of the RCA α- and β-isoforms and their abundance is relatively constant over a diurnal day/night cycle (**Figure 1A**). In contrast, phosphorylation of RCA at the Thr-78 site, as detected by immunoblotting with sequence- and modification-specific antibodies (anti-pT78 antibodies; **Figure 1A**), was only observed at night and both isoforms were equally phosphorylated. Full blots showing the specificity of these custom antibodies are presented in **Supplementary Figure S1**; apart from the α- and β-isoforms of RCA, only the anti-pT78 antibodies reacted with an additional off-target band (designated by the asterisk) that provides a useful loading control. Phosphorylation of Thr-78 was also triggered by exposure to low light, which resulted in partial phosphorylation compared to that observed after 1 h of complete darkness (**Figure 1B**). Thus, the light signal controlling RCA phosphorylation seems to not operate as an on–off switch, but rather is also responding to low irradiance with partial phosphorylation. In addition, the experiment presented in **Figure 1B** was performed in the middle of the day whereas the experiment in **Figure 1A** was performed where the dark period coincided with the normal night cycle. Thus is it is also clear that RCA phosphorylation is responding to instantaneous light conditions. The time course of phosphorylation and dephosphorylation at the Thr-78 phosphosite is presented in **Figure 1C** for plants transferred to darkness after 5 h of illumination. As shown, phosphorylation occurred rather slowly to reach a maximum value after 1 h of darkness and both RCA isoforms responded in similar fashion. In contrast, dephosphorylation upon transfer of plants to the light occurred rapidly and was nearly complete within 5 min.

**FIGURE 2:**
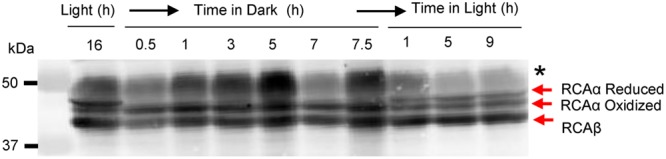
***In vivo* oxidation of the RCA α-isoform in the dark occurs rapidly and involves an intramolecular disulfide bond.** Leaves were harvested at the indicated times and subjected by non-reducing SDS-PAGE followed by immunoblotting with anti-RCA antibodies. The bands corresponding to reduced- and oxidized-RCAα and the single RCAβ isoform are indicated; the black asterisk identifies the RbcL protein, which shows increased antibody reaction when electrophoresis is performed under non-reducing conditions.

In *Arabidopsis*, RCA is well known to be regulated by reversible disulfide bond formation between Cys-451 and Cys-470 located in the C-terminus that is unique to the α-isoform (Zhang and Portis, 1999). We reasoned that because the disulfide was intra- rather than inter-molecular, the redox modification may be observed as faster migration of the oxidized α-isoform on non-reducing SDS-PAGE. This has been noted in the past with other redox-regulated enzymes (Pollitt and Zalkin, 1983) and is attributed to the more compact structure of the oxidized polypeptide compared to the reduced polypeptide. As shown in **Figure 2**, two bands corresponding to RCAα could be resolved from leaves harvested in the light, whereas only a single band with faster migration was observed in leaves harvested in the dark. The migration of the β-isoform was unaffected by these treatments consistent with the notion that it does not contain redox-sensitive sulfhydryl groups (Zhang and Portis, 1999). It is important to note that inter-molecular disulfides of the α-isoform were not observed (see **Supplementary Figure S2**). However, blots prepared from non-reducing gels invariably showed greater non-specific reaction of the ∼50-kDa Rubisco large subunit (RbcL) protein (identified with the black asterisk in **Figure 2** and **Supplementary Figure S2**).

### Role for Stromal Redox Status in Control of RCA Phosphorylation

**FIGURE 3:**
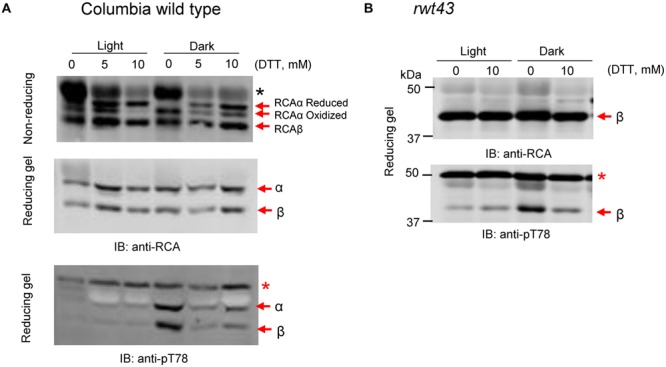
**Feeding DTT to leaves inhibits the dark-induced phosphorylation of RCA at the Thr-78 site.** Leaves were vacuum infiltrated in water containing 0, 5, or 10 mM DTT followed by 1 h in the light or dark. Extracts were subjected to non-reducing or reducing SDS-PAGE as indicated and blots were probed with anti-RCA or anti-pT78 antibodies. **(A)** *Arabidopsis* Col wild type plants with both RCA isoforms, subjected to SDS-PAGE under reducing or non-reducing conditions as indicated. **(B)** Transgenic rwt43 plants expressing only the RCAβ isoform, electrophoresed under reducing conditions only. In each blot, the bands corresponding to the α- and β-isoforms are indicated. The red asterisk in the anti-pT78 blots indicates an off-target signal that serves as a loading control, and the black asterisk in **(A)** identifies the RbcL protein that shows increased antibody reaction when electrophoresis is performed under non-reducing conditions.

To determine if stromal oxidation is the factor that triggers phosphorylation of RCA, wild type *Arabidopsis* or transgenic plants expressing just the non-redox regulated 43-kDa RCAβ isoform were examined for responses to exogenous DTT. In the experiment presented in **Figure 3**, leaves were vacuum infiltrated in water containing 0, 5, or 10 mM DTT prior to incubation for 1 h in the light or dark as indicated. Leaves were harvested at the end of light or dark treatment, and extracts were prepared and fractionated by non-reducing or reducing SDS-PAGE as indicated prior to immunoblot analysis. In wild type *Arabidopsis* leaf extracts analyzed by non-reducing SDS-PAGE, three bands of RCA protein were detected, including two corresponding to the reduced and oxidized forms of RCAα and one band corresponding to RCAβ (**Figure 3A**, middle). Incubation of leaves in the light with increasing concentrations of the reductant DTT promoted reduction of the intramolecular disulfide bond in RCAα such that at 10 mM DTT only a single RCAα band was observed that corresponds to the slower migrating reduced isoform. In the dark, control leaves (0 mM DTT) had a single α-isoform band corresponding to the oxidized, faster migrating form, but in the presence of DTT the reduced RCAα subunit was restored (**Figure 3A**). It is known that DTT can reduce oxidized thioredoxins (Buchanan et al., 1979) and that redox-regulated chloroplast enzymes can be reduced in the dark by DTT treatment of leaves (Kolbe et al., 2006); because RCAα is reductively activated by thioredoxin-f (Zhang and Portis, 1999), the results are indeed expected and confirm that the DTT treatment has altered the redox status of the chloroplast stroma. Analysis of the same samples on reducing SDS-PAGE detected only the single (reduced) RCAα and RCAβ bands (**Figure 3B**). Also as expected, phosphorylation of RCA Thr-78 was observed only in the dark in the control (-DTT) leaf sample where phosphorylation of both α- and β-isoforms was apparent. The most important result is that treatment of leaves with DTT in the dark largely prevented the phosphorylation, suggesting that oxidation of the stromal thioredoxin(s) was the trigger for phosphorylation of RCA (**Figure 3A**, bottom). The changes in RCA Thr-78 phosphorylation were also apparent when proteins were electrophoresed on non-reducing SDS-PAGE (not shown). In order to determine whether stromal redox was affecting phosphorylation of RCA indirectly as a result of reversible disulfide bond formation in the C-terminal extension of RCAα, we tested the effect of DTT treatment on leaves expressing only the non-redox regulated RCAβ isoform (**Figure 3B**). Because these plants only contain the non-redox regulated RCA isoform, electrophoresis was performed only under reducing conditions, where bands are sharper and non-specific antibody reactions are less pronounced (e.g., reaction of the antibodies with the RbcL band). As shown, phosphorylation of RCAβ at the Thr-78 site occurred in the darkened control leaf sample and was greatly reduced by 10 mM DTT. These results suggest that it is not the redox status of RCA itself that affects phosphorylation of the protein, but rather other steps in the process.

**FIGURE 4:**
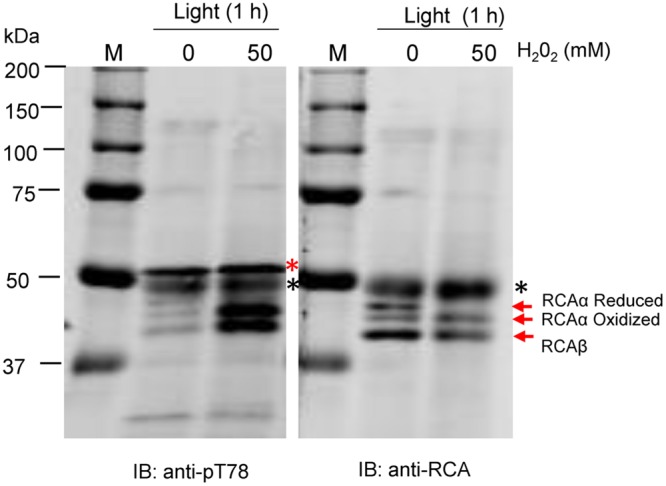
**Feeding H_2_O_2_ in the light causes RCAα oxidation and induces phosphorylation at the Thr-78 site.** SDS-PAGE was performed under non-reducing conditions and blots were probed with anti-RCA or anti-pT78 antibodies as indicated. The bands corresponding to the α- and β-isoforms of RCA are indicated. The red asterisk in the anti-pT78 blot indicates an off-target signal that serves as a loading control, and the black asterisk identifies the RbcL protein that shows increased antibody reaction when electrophoresis is performed under non-reducing conditions.

To further test the role of stromal redox, leaf segments were incubated in the light with and without H_2_O_2_ (**Figure 4**). In the absence of H_2_O_2_, both the reduced and oxidized forms of RCAα and a single band of RCAβ were detected by the anti-RCA antibodies after non-reducing SDS-PAGE. However, with 50 mM (**Figure 4**) and 100 mM (not shown) H_2_O_2_, in addition to the RCAβ band only a single band corresponding to oxidized RCAα was observed indicating that the Trx system had been oxidized by the exogenous supply of H_2_O_2_ in the light. Importantly, treatment with exogenous H_2_O_2_ resulted in phosphorylation of both α- and β-isoforms of RCA in the light, further supporting the notion that the light/dark control of phosphorylation is mediated by the plastid Trx system. In blots produced from non-reducing SDS-PAGE gels, increased reaction of antibodies with the RbcL protein was apparent with both the anti-pT78 and anti-RCA antibodies (**Figure 4**, marked with black asterisks), but the non-specific reaction did not interfere with visualization of the RCA isoforms in either blot.

### Plastid CK2α Phosphorylates RCA at the Thr-78 Site

**FIGURE 5:**
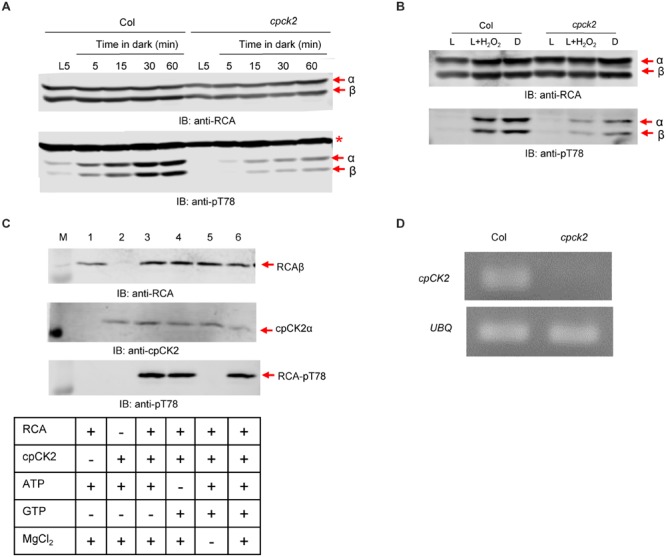
**Plastid CK2α is the major protein kinase responsible for phosphorylation of RCA at the Thr-78 site.** The *cpck2* mutant has dramatically reduced phosphorylation of RCA at the Thr-78 site following **(A)** transfer of plants from the light to dark or **(B)** treatment of leaves with H_2_O_2_ for 1 h in the light. **(C)** Phosphorylation of recombinant RCAβ with recombinant cpCK2α *in vitro* confirms the involvement of CK2α. In each blot, the bands corresponding to the α- and β-isoforms, as appropriate, are indicated. The red asterisk in the anti-pT78 blot in **(A)** indicates an off-target signal that serves as a loading control. **(D)** Semi-quantitative RT-PCR analysis of the T-DNA insertion line, GABI400A04. Primers complementary to sequences flanking the insertion site in the cpCK2 mRNA were used. Primers within the ubiquitin (UBQ) coding sequence were used as a positive control.

The sequence surrounding RCA-Thr78 is flanked by Asp residues suggesting that CK2 may be the requisite kinase. Chloroplasts are known to contain one member of the CK2 family known ascpCK2α, which is thought to be a major protein kinase in the plastid. To test the possible role of cpCK2α in RCA phosphorylation, we obtained a homozygous T-DNA mutant (GABI400A04) with the insert in the fifth exon of the gene that allowed us to test the function of this protein kinase *in vivo*. Indeed, disruption of *cpCK2α* substantially reduced phosphorylation of RCA at the Thr-78 site relative to wild type plants when leaves were darkened (**Figure 5A**), or when phosphorylation of RCA was induced in response to H_2_O_2_ feeding in the light (**Figure 5B**). These results suggest that cpCK2α is the major kinase involved in phosphorylation of RCA at Thr-78 *in vivo*. To test the role of cpCK2α further, we produced recombinant cpCK2α in *E. coli* for *in vitro* phosphorylation assays using recombinant RCAβ as the substrate. As shown in **Figure 5C**, recombinant cpCK2α-His_6_ phosphorylated recombinant RCAβ at the Thr-78 site *in vitro*, consistent with its suggested role *in vivo*. Phosphorylation of RCA was dependent upon cpCK2α and as is characteristic of the entire CK2 family (Niefind et al., 1999), cpCK2α required Mg^2+^ but could utilize ATP *or* GTP as co-substrate in the phosphorylation reaction. These results are consistent with the general notion that cpCK2α is the cognate kinase involved in RCA phosphorylation *in vivo*. Inactivation of the *cpCK2* gene in the T-DNA insertional line used in these experiments was tested using semi-quantitative reverse transcription (RT)-PCR. As shown in **Figure 5D**, the mRNA for *cpCK2* was present in wild type Columbia plants but was not present in the insertional mutant, confirming the knockout phenotype.

**FIGURE 6:**
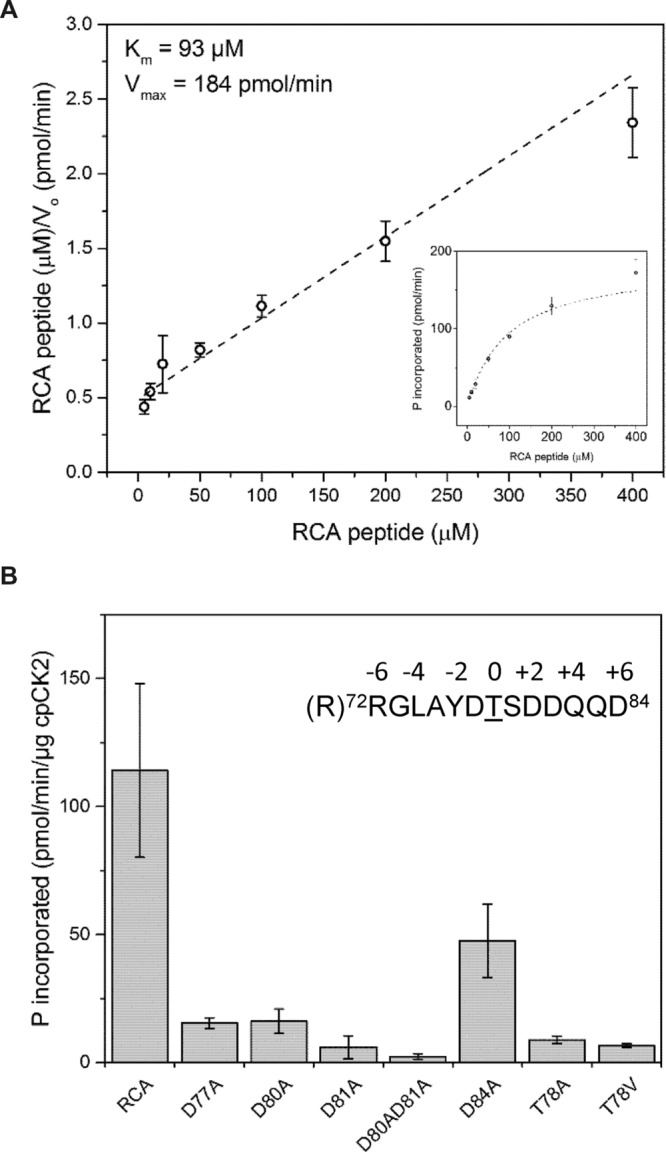
**Recombinant cpCK2 phosphorylates a Thr78 synthetic peptide. (A)** Hanes–Woolf plot for determination of kinetic constants for phosphorylation of the RCA peptide (sequence shown in panel **B**) by cpCK2-His_6_. Michaelis–Menten plot of the same data is shown (inset). Open circles represent mean with 95% CI of six technical replicates for each concentration of substrate peptide. Dashed line represent linear fit of the data used for determination of *K*_m_ and V _max_. Interpolated values for *K*_m_ and V _max_ are shown. **(B)** Characterization of a cpCK2 recognition motif using variants of the RCA peptide (sequence RRGLAYDTSDDQQD). Synthetic peptides contained Ala substitutions for acidic residues (Asp) surrounding the phosphoacceptor Thr-78 or, Ala or Val substitutions for the phosphoacceptor Thr-78. Specific activity was measured using 100 μM of wild type (RCA) or peptide variants as indicated. Bars represent mean specific activity (pmol min^-1^μg^-1^ cpCK2) with 95% CI of six technical replicates for each peptide variant.

The synthetic peptide RRGLAYDTSDDQQD, based on the sequence surrounding Thr-78 (underlined) but with an additional Arg residue added to the N-terminus, was readily phosphorylated by cpCK2α (**Figure 6A**). Kinetics were generally hyperbolic (**Figure 6A** inset) with an apparent Km of 93 μM, which is consistent with efficient targeting of RCA Thr-78 as a phosphosite. In order to determine which amino acids surrounding the Thr-78 phosphosite might contribute to recognition by cpCK2α, variants of the base peptide identified above were prepared with Ala substitutions at selected single positions. Substitution of the Thr, designated as position 0, with an Ala residue (the T78A peptide) strongly reduced phosphorylation consistent with the notion that phosphorylation on the peptide substrate occurred primarily on the Thr residue and in its absence, the Ser residue at position +1 could not substitute to a significant extent. Likewise, substitution of Thr at position 0 with Val (T78V peptide) had a generally similar effect and did not increase phosphorylation at the adjacent Ser residue. All of the individual Asp-to-Ala substitutions had a strong inhibitory effect on peptide phosphorylation, with substitution at position +3 (the D81A peptide) having the greatest single effect. However, the acidic residues at -1 (D77A peptide) and +2 (D80A peptide) were also very important, followed by the Asp at position +6 (D84A peptide). These results are generally consistent with the notion that CK2 kinases target specific phosphosites based on the acidic residues at positions +3 (most critical), +1, and +2 (Meggio and Pinna, 2003).

### Evidence for Cross Talk between Phosphorylation and Redox Control of RCA *In Vivo*

**FIGURE 7:**
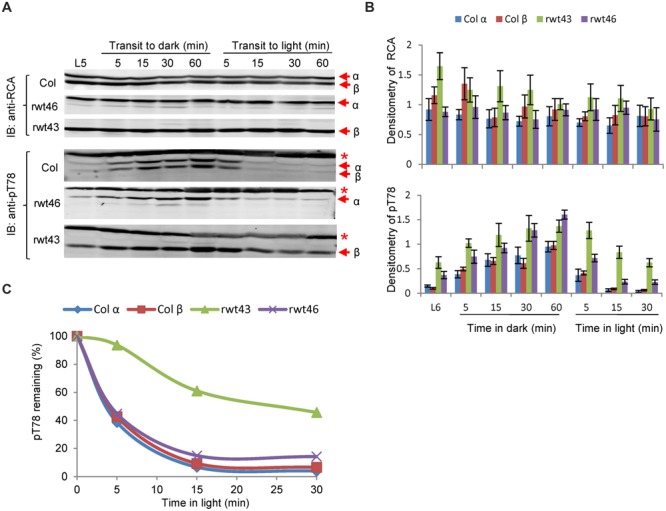
**Evidence for cross talk between phosphorylation of Thr78 and redox control of RCAα. (A)** Immunoblot analysis of RCA protein and phosphoThr-78 in wild type *Arabidopsis* and the rwt43 and rwt46 transgenic plants following a midday transfer to darkness and return to light. The experiment started 5 h after the beginning of the photoperiod (L5 sample). The red asterisk in the anti-pT78 blots indicates an off-target signal that serves as a loading control. **(B)** Densitometric analysis of the immunoblots in **(A)** and two additional replicate experiments. Values are means ± standard error of the mean. **(C)** Relative dephosphorylation of phosphoThr-78 following transfer from dark to light conditions. Mean values from **(B)** were normalized to the phosphoThr-78 level after 60 min of darkness. The key point to note is that dephosphorylation of the RCAβ protein in the rwt43 plants [green bars in **(B)** and green line in **(C)**] was markedly slower upon transfer from dark to light compared to the other genotypes.

Because redox regulation of RCAα and phosphorylation of both isoforms occurs in a light/dark-regulated manner, it was of interest to determine whether there might be some direct or indirect cross talk between the two post-translational modifications. To investigate this, we examined the kinetics of RCA phosphorylation in wild type and transgenic *Arabidopsis* plants expressing only the redox-regulated α-isoform (rwt46) or non-redox regulated β-isoform (rwt43). As shown in **Figure 7**, wild type and rwt46 plants were similar in that phosphorylation of the Thr-78 site occurred relatively slowly upon transfer to darkness, but the phosphosite was rapidly dephosphorylated upon transfer to light. In contrast, the rwt43 plants maintained a higher level of phosphoThr-78 after 5 h of light (the ‘L5’ sample in **Figures 7A,B**), and dephosphorylation upon transfer from dark to light was considerably slower compared to wild type and rwt46 plants. This is apparent by visual inspection of the immunoblots in **Figure 7A** and was confirmed by the densitometric analysis shown in **Figure 7B**. Differences in the rate of dephosphorylation of phospho-Thr78 following transfer from dark to light conditions were also apparent when values were normalized to the phosphorylation level after 60 min of darkness (**Figure 7C**). Collectively, these results suggest that the C-terminus of the α-isoform may somehow facilitate dephosphorylation of phosphoThr-78.

### Function of Phosphorylation of RCA

**FIGURE 8:**
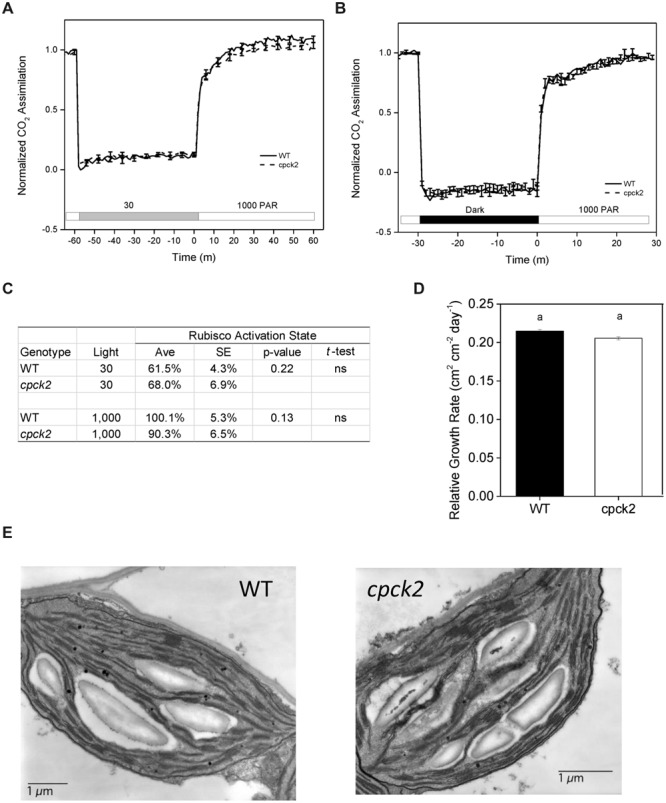
**The cpck2 mutant is generally similar to wild type *Arabidopsis* suggesting that phosphorylation of RCA at the Thr-78 site is not playing an essential role *in vivo*.** Induction kinetics of photosynthesis following pretreatment in **(A)** low light or **(B)** darkness. **(C)** Rubisco activation state after exposure of leaves for 60 min to low or high light as in the experiment in **(A)**. **(D)** Relative plant growth rate in short days determined based on leaf area expansion. **(E)** Chloroplast structure showing normal granal development and accumulation of starch granules at the end of the day.

As one approach to study the functional role of phosphorylation *in vivo*, we examined the induction of leaf photosynthesis by gas exchange following changes in irradiance with wild type *Arabidopsis* and the *cpck2* knockout mutant that has strongly reduced phosphorylation at the Thr-78 site (**Figure 5A**). It is generally accepted that RCA activity controls the rate of induction of photosynthesis following transfer from low to high light (Mott and Woodrow, 2000; Carmo-Silva and Salvucci, 2013). Therefore, if phosphorylation of Thr-78 is important in regulating RCA activity during light transitions, preventing phosphorylation of RCA by genetic removal of the requisite protein kinase, cpCK2α should impact induction kinetics of photosynthesis. Nearly identical photosynthetic induction kinetics were observed for wild type and the *cpck2* knockout mutant when plants were transferred from low light (**Figure 8A**) or darkness (**Figure 8B**) to high light. Additionally, *in vitro* Rubisco activity assays measured under similar conditions revealed deactivation of Rubisco in leaves of wild type plants and the *cpck2* knockout mutant at low light compared to high light and the differences between the genotypes under each light condition were not statistically significant based on the *t*-test (**Figure 8C**). These results strongly suggest that at least in wild type *Arabidopsis* plants expressing both RCA isoforms, phosphorylation of RCA does not play an essential and non-redundant role relative to redox regulation in deactivation of RCA activity at low light.

As might be expected based on similar photosynthetic characteristics, the *cpck2* knockout mutant had a nearly identical relative growth rate compared to wild type plants (**Figure 8D**) and chloroplast structure appeared normal with prominent starch granules present at the end of the day similar to wild type plants (**Figure 8E**).

## Discussion

The results of the present study add substantially to our understanding of the modification of RCA by phosphorylation at the Thr-78 position. This phosphosite is well established in the literature (Reiland et al., 2009, 2011; Aryal et al., 2012; Meyer et al., 2012; Wang et al., 2013) and there is compelling evidence for light–dark regulation of this phosphorylation (Reiland et al., 2009; Boex-Fontvieille et al., 2013). Because Thr-78 resides in the N-domain of RCA that is essential for its interaction with Rubisco (Salvucci et al., 1994; Stotz et al., 2011), a regulatory role for this phosphorylation event seems reasonable if not likely (Boex-Fontvieille et al., 2013). A major finding of the present study is that, unlike redox regulation, phosphorylation is not essential for control of Rubisco activity in response to changes in irradiance. However, the possibility that phosphorylation plays a regulatory role in the absence of redox control remains to be tested in the future. Although a functional role has not yet been uncovered, our results do provide additional foundational information about the nature of phosphorylation at Thr-78 and factors controlling it.

### Light/Dark Control of RCA Phosphorylation Is Mediated by Stromal Redox

Using custom antibodies to detect phosphorylation at the Thr-78 site by immunoblotting allowed us to determine that phosphorylation occurs on both RCAα and RCAβ in a light-regulated manner. Light was not an on–off switch, but rather a fine-tuning mechanism so that phosphorylation was partially induced at low irradiance (20 μmol photons m^-2^ s^-1^) compared to complete darkness (**Figure 1B**). Dark-induced phosphorylation occurred regardless of whether the dark treatment coincided with the normal night period or was imposed during the middle of the day. Stromal redox status may be an important factor because feeding DTT that can chemically reduce Trx proteins largely blocked the dark-induced phosphorylation of RCA (**Figure 3A**). Conversely, feeding H_2_O_2_ induced phosphorylation of RCA in the light (**Figure 4**). Therefore, oxidized Trx in the stroma appears to be the trigger for dark-induced phosphorylation of RCA at the Thr-78 site under normal conditions. Conceivably, the impact of oxidized Trx on inducing phosphorylation could be on RCA itself or could involve other components. Two lines of evidence suggest that the effect is not directly on RCA. First, phosphorylation of RCAβ in the rwt43 plants (**Figure 3B**) was as sensitive to DTT inhibition as wild type plants. Because the β-isoform lacks the redox Cys pair of the α-isoform, this result strongly suggests that redox changes in the RCA protein itself are not involved in the regulation of Thr-78 phosphorylation. A second point to note is that phosphorylation of RCA in the dark was relatively slow compared to formation of the intramolecular disulfide bond in the α-isoform. The latter was complete within 30 min (**Figure 2**) and within 10 min in other experiments (data not shown) whereas phosphorylation usually required nearly 60 min to reach a maximum (**Figures 1A, 4**, and **6**). Collectively our results are most consistent with the notion that dark conditions result in thioredoxin oxidation (Wittenberg and Danon, 2008), which would be expected to trigger formation of the disulfide bond in the C-terminal extension of RCAα that confers ADP inhibition to the oligomeric protein, and as a result RCA activity is inhibited and Rubisco deactivates. Independently, Trx oxidation either activates cpCK2α and/or inhibits the protein phosphatase, and as a result RCA is phosphorylated at the Thr-78 site. Another formal possibility is that stromal redox, or some aspect related to it, affects RCA oligomeric structure or RCA subunit interactions in such a way that phosphorylation or dephosphorylation are altered.

We observed that the RCAβprotein, in the rwt43 transgenic plants that lack the α-isoform, was dephosphorylated more slowly upon transfer from dark to light compared to wild type plants or rwt46 transgenic plants expressing only the α-isoform (**Figure 7**). While the causal basis for this observation is not clear, it is possible that the C-terminal extension of RCAα provides a docking site for the protein phosphatase. Alternatively, the C-terminus may affect conformation of the N-domain of RCA impacting accessibility of phosophoThr-78 to the protein phosphatase(s). Finally, it is possible that RCAβ oligomerizes *in vivo* somewhat differently compared to RCAα (or the mixture of isoforms found in wild type plants) such that the substrate protein (RCA phosphoThr-78) is less accessible to the protein phosphatases. Indeed, differences in oligomerization *in vitro* between the two isoforms have been observed (Hasse et al., 2015). Distinguishing these possibilities will require further study, and may be facilitated by identification of the protein phosphatase. However, at this point we can conclude that there are likely several ways in which redox and phosphorylation of RCA may be related. The first is that the redox status of the chloroplast stroma directly controls formation of the regulatory disulfide in the C-terminus of RCAα and in some manner increases net RCA phosphorylation. Thus, the two post-translational modifications are coordinated by the same redox signal that likely involves oxidized thioredoxins. Secondly, the redox-active C-terminus of RCAα affects the steady-state phosphorylation of RCA and specifically the rate of dephosphorylation upon transfer of plants from dark to light. Conceivably, this may involve access of the protein phosphatase to the phosphosite, however, as discussed above the molecular basis for this interaction remains to be elucidated.

### Plastid CK2α Is the Requisite Protein Kinase Phosphorylating RCA Thr-78

Our studies provide strong support for the notion that cpCK2α plays a major role in phosphorylation of RCA. As noted in an earlier study (Boex-Fontvieille et al., 2013), the sequence surrounding Thr-78 contains several flanking acidic residues and therefore could be phosphorylated by a member of the CK2 family. Chloroplasts contain a single, nuclear-encoded CK2α subunit (Bayer et al., 2012), and Motif-X analysis of chloroplast phosphoproteins revealed several overrepresented phosphorylation motifs including pSDxE (Reiland et al., 2009), which is the canonical CK2 motif (Meggio and Pinna, 2003). Chloroplast protein phosphosites have also been used to develop peptide microarrays for *in vitro* phosphorylation experiments to identify the *in vivo* substrates of cpCK2α (Schonberg et al., 2014) using recombinant protein as well as native kinase preparations. In their studies, cpCK2α preferred Ser over Thr as the phospho acceptor in sequences with acidic residues at +1 and +3, and to a lesser extent at +2 and +5. A peptide based on the sequence surrounding RCA Thr-78 was phosphorylated but only “weakly” by recombinant and native cpCK2α (Schonberg et al., 2014), leaving somewhat open the question of whether cpCK2α is a candidate kinase for phosphorylation of RCA Thr-78. Analysis of the *cpck2* mutant establish that cpCK2α is the major kinase responsible for RCA phosphorylation *in vivo* (**Figures 5A,B**) and studies with recombinant protein confirmed that RCA was a good substrate for cpCK2α *in vitro* (**Figure 5C**).

Our studies with recombinant cpCK2α and synthetic peptide substrates provide new insights regarding the targeting of *Arabidopsis* RCA Thr-78. It is generally recognized that CK2 kinases target their substrates based on the positioning of acidic residues, especially C-terminal to the phosphosite. Setting the phosphorylated Ser or Thr as position 0, acidic residues at +1 and +3 in particular are considered to be most important (Meggio and Pinna, 2003; Schonberg et al., 2014). In *Arabidopsis* RCA, Thr-78 is surrounded by acidic residues at the –1, +2, +3, and +6 positions and our studies with synthetic peptide variants as substrates determined that all function as positive recognition elements. In particular, the Asp residues at –1, +2, and +3 were most important, with the Asp at +3 almost essential for phosphorylation (**Figure 6B**). Thus, within the sequence context of *Arabidopsis* RCA, Thr-78 was ideally positioned for recognition and phosphorylation by cpCK2α. Interestingly, an acidic residue at the +1 position was not present indicating that motifs can display some flexibility.

Given the role of cpCK2α in phosphorylation of various components involved in chloroplast transcription (Baginsky et al., 1999; Ogrzewalla et al., 2002; Pfannschmidt and Liere, 2005; Turkeri et al., 2012) it is indeed surprising that a knockout mutant grows similar to wild type plants in terms of growth rate (**Figure 8D**) and with normal chloroplast structure (**Figure 8E**). Previous studies with a *cpck2α* knockout mutant determined that ABA sensitivity and thermotolerance were reduced, and suggested that cpCK2α functions as a positive regulator of nuclear gene expression (retrograde signaling) in response to heat and ABA (Wang et al., 2014). In future studies, it will be interesting to determine the impact of the *cpck2α* knockout on the chloroplast phosphoproteome, and specifically the phosphorylation status of key transcriptional and retrograde signaling components. While phosphorylation of RCA is substantially reduced in the *cpck2α* mutant, it is conceivable that other casein kinase-like proteins may sufficiently compensate to maintain phosphorylation of other substrate proteins thereby minimizing the phenotypic impact.

### Phosphorylation Plays Little or No Role in Regulation of RCA Activity *In Vivo* under Normal Conditions

Attempts to identify a functional role for phosphoThr-78 have been unsuccessful. Gas exchange studies with the *cpck2* knockout mutant that has strongly reduced phosphorylation at the Thr-78 site (**Figure 5**), documented similar rates of photosynthetic induction and Rubisco activation state compared to wild type plants following transition from darkness or low light to high light (**Figures 8A–C**). It should be noted that while the *in vivo* activation states under high and low light agree well with some previous work in *Arabidopsis* (Carmo-Silva and Salvucci, 2013), they are higher than those found in other work (Scales et al., 2014). These differences could be attributed to the higher incubation temperatures used in those studies (Scales et al., 2014; 30°C instead of 25°C). Importantly, our results comparing wild type plants and the *cpck2* knockout suggest that Rubisco deactivation occurred equivalently in the two genotypes in low light and therefore, phosphorylation is not required for the down regulation of RCA activity when normal redox regulation is occurring. The potential for a role of phosphorylation under other conditions, such as under stress, or in the absence of the redox-regulated α-isoform, are worth exploring in the future.

## Conclusion

The central regulator of post-translational modification of *Arabidopsis* RCA is the redox status of the chloroplast stroma. Under normal dark conditions, plastid Trx proteins are largely oxidized while other components of the stromal redox system such as the glutathione pool and ascorbate remain highly reduced (Foyer and Noctor, 2015). Oxidation of Trx proteins results in the rapid formation of regulatory disulfides in many stromal proteins including RCAα. The oxidation of RCAα enhances ADP inhibition and therefore under physiological conditions, RCA activity is strongly inhibited and Rubisco deactivation results. Oxidation of thioredoxins somehow also triggers the phosphorylation of RCA at the Thr-78 site although the mechanism remains unclear and will be the focus of future studies. In addition, the impact of phosphorylation on RCA activity is uncertain, although current results suggest that at least in the presence of redox regulation, phosphorylation does not play an essential role in the regulation of Rubisco activity. The potential for phosphorylation of RCA to impact other properties such as ATPase activity, RCA degradation, thylakoid membrane association or heat stability of RCA, will be investigated in future studies. Furthermore, phosphorylation could contribute to control of RCA activity under conditions where redox changes were not occurring. Alternatively, RCA could have a presently unrecognized function that does not involve Rubisco activation and that function may be activated or otherwise regulated by phosphorylation. The use of the *cpck2* mutant and transgenic plants expressing the T78A directed mutant will be useful in searching for potential functions under a variety of conditions.

## Author Contributions

All of the authors contributed to the planning and interpretation of experiments, and commented on the manuscript at all stages. SK performed the immunoblot experiments; KB performed the peptide kinase assays; and BW performed the gas exchange experiments and relative growth rate analysis and supervised the activation state assays.

## Conflict of Interest Statement

The authors declare that the research was conducted in the absence of any commercial or financial relationships that could be construed as a potential conflict of interest.

## Supplementary Material

FIGURE S1 **Full panels of the trimmed immunoblots presented in Figure 1A showing the specificity of the anti-RCA (A) and anti-pT78 (B) antibodies used in this study**. The off-target band (marked with the asterisk) that migrates just above the Rubisco large subunit (RbcL) protein and reacts with the anti-pT78 antibodies provides a useful loading control.

FIGURE S2 **Full panel of the trimmed immunoblot presented in Figure 2 showing that no inter-subunit disulfide bonds with RCAα were apparent when non-reducing SDS-PAGE gels were used to fractionate proteins prior to immunoblot analysis.** The blot was intentionally visualized at a higher sensitivity than used in **Figure 2** to help distinguish any low abundance, higher molecular weight forms that might represent formation of intermolecular disulfides.
