# Supplementary material for: The Plastid Casein Kinase 2 Phosphorylates Rubisco Activase at the Thr-78 Site but Is Not Essential for Regulation of Rubisco Activation State
Source: Front Plant Sci. 2016 Mar 31;7:404. doi: 10.3389/fpls.2016.00404 (PMC4814456; doi:10.3389/fpls.2016.00404)
Supplement: Supplementary file 1 [file Table_1.DOCX]

Supplementary Table S1. Oligonucleotide primers used to generate the pET28a:cpCK2 expression clone.

| Name | Restriction site | Sequence^a^ |
| --- | --- | --- |
| cpCK2∆NcoI | n/a | CGTGGGCAGACATAGCCGGAAGCCtTGGACAAAGTTCATCAATTCTG |
| cpCK2∆NcoIrc | n/a | CAGAATTGATGAACTTTGTCCAaGGCTTCCGGCTATGTCTGCCCACG |
| cpCK2NcoIF | NcoI | ATCGCCATGGCTTCTCTTTACCGTCAAC |
| cpCK2XhoIR^b^ | XhoI | ATCGCTCGAGCTGGCTGCGCGGCGTACG |
| a, lower case letter indicates nucleotide base targeted for mutagenesis; underlined nucleotides indicate translational start site  b, oligonucleotide primer lacks the native stop codon for in-frame fusion with a C-terminal 6xHis coding sequence | | |
