# Supplementary material for: The Plastid Casein Kinase 2 Phosphorylates Rubisco Activase at the Thr-78 Site but Is Not Essential for Regulation of Rubisco Activation State
Source: Front Plant Sci. 2016 Mar 31;7:404. doi: 10.3389/fpls.2016.00404 (PMC4814456; doi:10.3389/fpls.2016.00404)
Supplement: Supplementary file 2 [file Table_2.DOCX]

Supplementary Table S2. Synthetic peptide substrates used to assay cpCK2-His_6_ substrate phosphorylation.

| Name | Sequence^a^ | Molecular weight (Da) |
| --- | --- | --- |
| RCA | RRGLAYDTSDDQQD | 1639.64 |
| D77A | RRGLAY*A*TSDDQQD | 1595.63 |
| T78A | RRGLAYD*A*SDDQQD | 1609.62 |
| T78V | RRGLAYD*V*SDDQQD | 1637.67 |
| D80A | RRGLAYDTS*A*DQQD | 1595.63 |
| D81A | RRGLAYDTSD*A*QQD | 1595.63 |
| D80AD81A | RRGLAYDTS*AA*QQD | 1551.62 |
| D84A | RRGLAYDTSDDQQ*A* | 1595.63 |
| a, underlined residue indicates threonine-78 phosphoacceptor; italicized residue indicates substituted residue | | |
