# Supplementary figures and images for: The Plastid Casein Kinase 2 Phosphorylates Rubisco Activase at the Thr-78 Site but Is Not Essential for Regulation of Rubisco Activation State
Source: Front Plant Sci. 2016 Mar 31;7:404. doi: 10.3389/fpls.2016.00404 (PMC4814456; doi:10.3389/fpls.2016.00404)

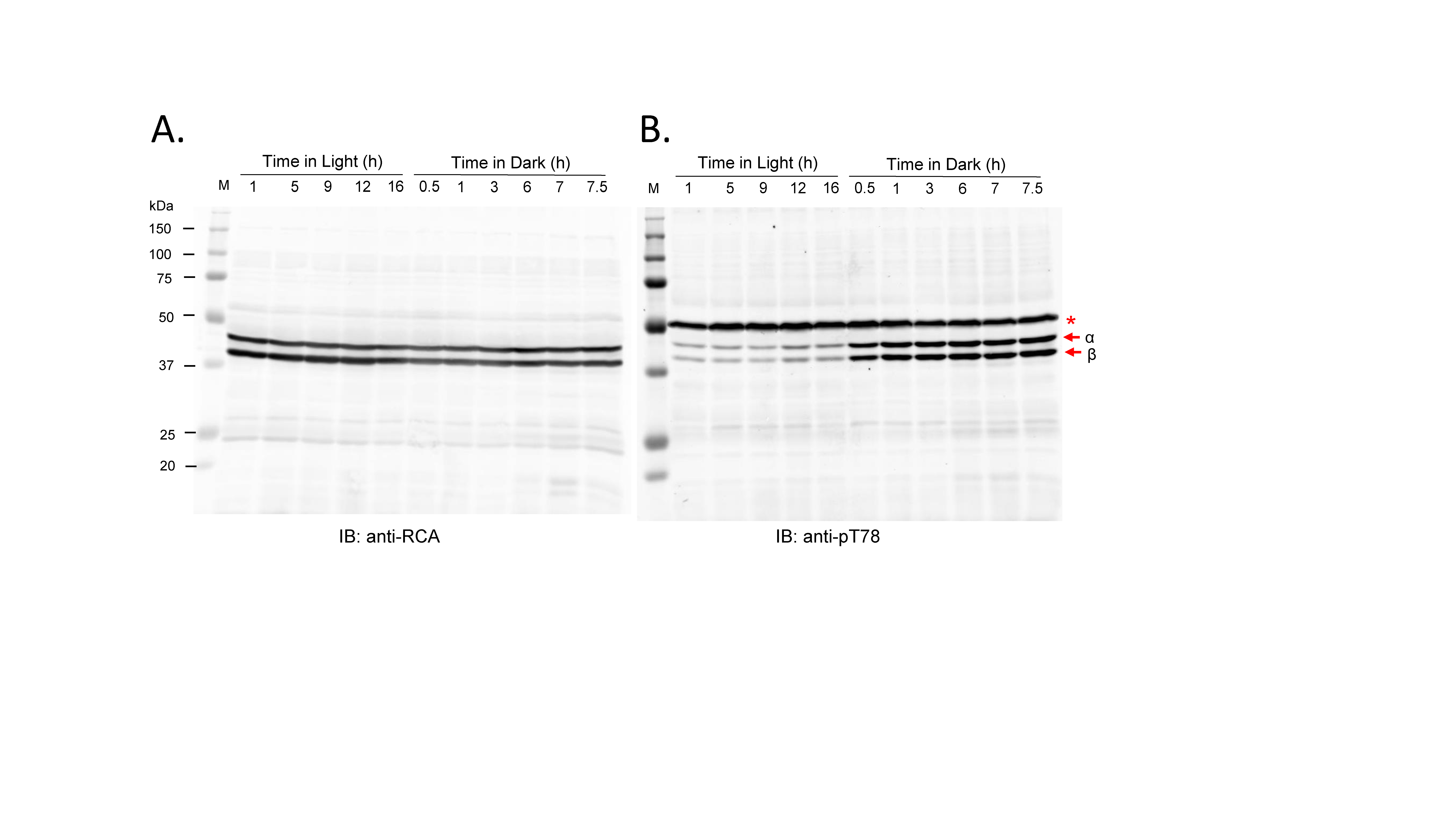

Supplement: FIGURE S1 — Full panels of the trimmed immunoblots presented in Figure 1A showing the specificity of the anti-RCA (A) and anti-pT78 (B) antibodies used in this study. The off-target band (marked with the asterisk) that migrates just above the Rubisco large subunit (RbcL) protein and reacts with the anti-pT78 antibodies provides a useful loading control. [file Image_1.TIF]

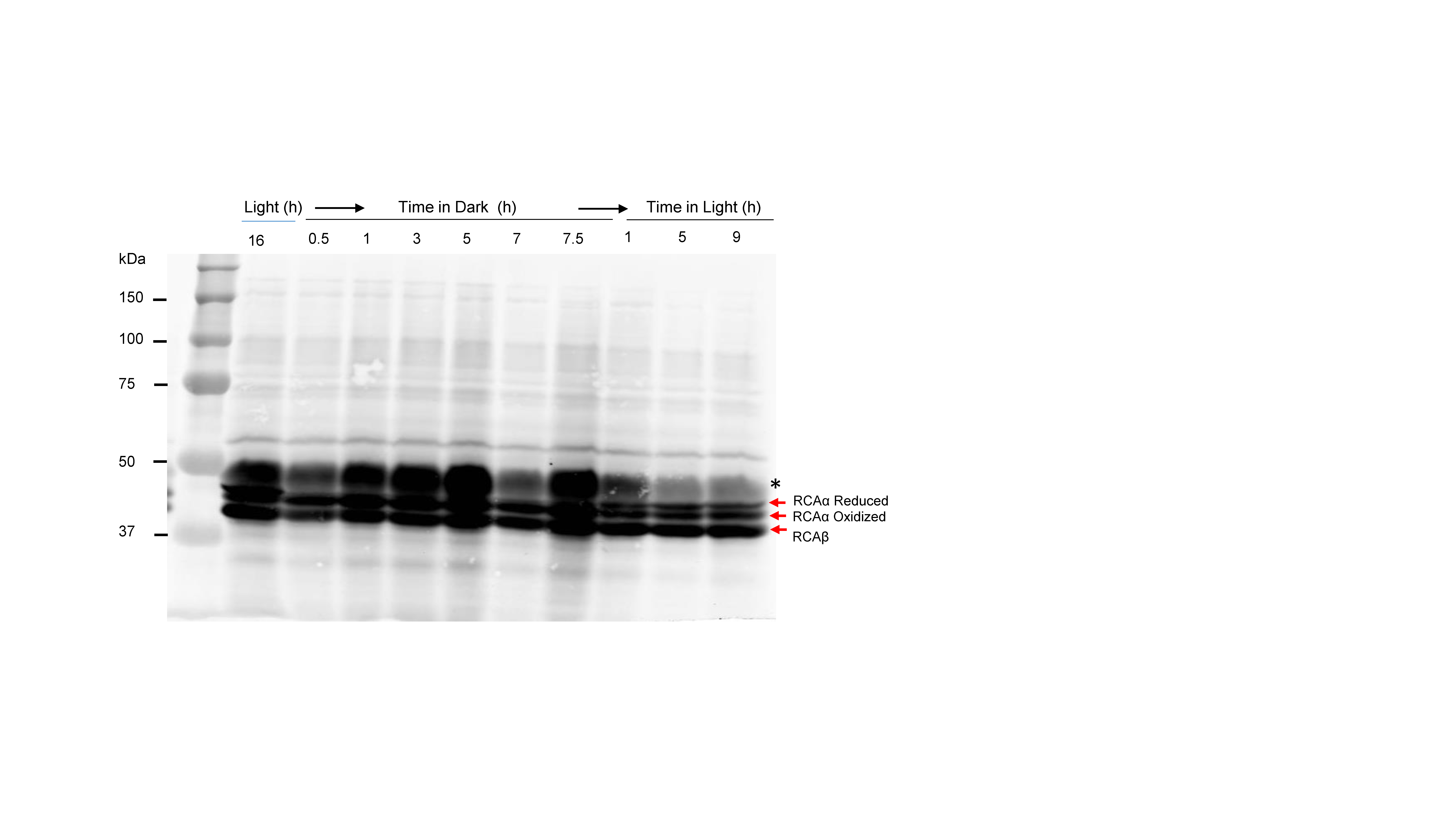

Supplement: FIGURE S2 — Full panel of the trimmed immunoblot presented in Figure 2 showing that no inter-subunit disulfide bonds with RCAα were apparent when non-reducing SDS-PAGE gels were used to fractionate proteins prior to immunoblot analysis. The blot was intentionally visualized at a higher sensitivity than used in Figure 2 to help distinguish any low abundance, higher molecular weight forms that might represent formation of intermolecular disulfides. [file Image_2.TIF]
